# Unlocking the Therapeutic Potential of *Trigonella foenum-graecum* and *Trigonella corniculata* Against High-Fat-Diet-Induced Hyperlipidemia: Antioxidant and Histopathological Evidence

**DOI:** 10.3390/medicina61122130

**Published:** 2025-11-28

**Authors:** Rabiya Shamim, Khurram Afzal, Asad Abbas, Muhammad Tauseef Sultan, Talha Bin Iqbal, Abdul Malik, Nikhat J. Siddiqi, Mohammad Shamsul Ola, Abdul Aziz Alamri, Abeeb Oyesiji Abiodum, Bipindra Pandey

**Affiliations:** 1 Department of Human Nutrition, Faculty of Food Science and Nutrition, Bahauddin Zakariya University, Multan 60800, Pakistan; rabiyashamim60@gmail.com (R.S.);; 2 Department of Food Science and Technology, Faculty of Food Science and Nutrition, Bahauddin Zakariya University, Multan 60800, Pakistan; 3 Department of Pharmaceutics, College of Pharmacy, King Saud University, Riyadh 11451, Saudi Arabia; amoinuddin@ksu.edu.sa; 4 Department of Medical Surgical Nursing, College of Nursing, King Saud University, Riyadh 11451, Saudi Arabia; nikhat@ksu.edu.sa; 5 Department of Biochemistry, College of Science, King Saud University, Riyadh 11451, Saudi Arabia; 6 121 Health Research Centre, James Lee Hall Extension, Southern University A&M College, Baton Rouge, LA 70813, USA; 7 Department of Pharmacy, Madan Bhandari Academy of Health Sciences, Hetauda 44107, Nepal

**Keywords:** *Trigonella foenum-graecum*, *Trigonella corniculata*, high-fat-diet-induced hyperlipidemia, antioxidant activity, hypolipidemic potential, in vivo study, histopathological analysis

## Abstract

*Background and Objectives*: This study investigated the antioxidant, lipid-lowering, and hepatoprotective effects of two fenugreek seed varieties, *Trigonella foenum-graecum* (TFG) and *Trigonella corniculata* (TC), and analyzed their bioactive potential using various solvents, doses, and biochemical parameters. *Materials and Methods:* Antioxidant analyses, including ferric-reducing antioxidant power (FRAP), total phenolic content (TPC), and 2,2-Diphenyl-1-picrylhydrazyl (DPPH) assays, were conducted, and interventional studies were performed on rats divided into groups receiving disease + standard basal diet (G_0_), standard basal diet only (G_1_), and disease + standard basal diet supplemented with TC or TFG at 400 mg/kg/day (G_2_, G_3_) and 800 mg/kg/day (G_4_, G_5_). Biochemical blood tests assessing lipid profiles and liver function parameters, coupled with histopathological examination of the liver and heart tissues, were also performed. *Results:* Antioxidant assessments indicated that TFG exhibited greater free radical scavenging ability, higher total phenolic content, and stronger ferric-reducing power than TC did. In the in vivo experiments, both TFG and TC significantly enhanced lipid profiles by reducing total cholesterol, low-density lipoprotein cholesterol (LDL-c), very-low-density lipoprotein cholesterol VLDL-c, and triglycerides while boosting high-density lipoprotein cholesterol (HDL-c) levels (*p* < 0.001). Liver function tests indicated significant decreases in bilirubin, alanine aminotransferase (ALT), aspartate aminotransferase (AST), and alkaline phosphatase (ALP) levels with dose and plant effects, particularly at 800 mg/kg (G_5_). Histopathological examination revealed that TFG at a dose of 800 mg/kg led to an almost normal liver structure and intact myocardial fibers with minimal inflammation, whereas TC groups displayed slight vacuolation of hepatocytes and some inflammatory responses. *Conclusions:* In conclusion, TFG shows the superior functional food properties of TFG in managing oxidative stress and hyperlipidemia in comparison to TC. Future studies should aim to elucidate the molecular mechanisms, optimize dosing regimens, and evaluate long-term safety and efficacy to support clinical applications.

## 1. Introduction

Dyslipidemia, a significant risk factor for cardiovascular diseases, is one of the disorders that can adversely affect quality of life [1,2]. In Pakistan, the prevalence of dyslipidemia and its related disorders is notably high. A survey conducted in Pakistan in 2023 revealed that approximately 92.26% of the studied CAD patients exhibited a dual or triple pattern of dyslipidemia, known as mixed dyslipidemia [3,4,5]. Numerous drugs are employed in managing and treating dyslipidemia and other ailments; however, these medications often come with side effects [6,7]. As lifestyle-related disorders, such as dyslipidemia, become more prevalent, there is growing interest in natural substances that have minimal side effects [8]. Fenugreek is known to contain a variety of bioactive components with therapeutic potential. Yet, the therapeutic potential of one variety, *Trigonella corniculata*, remains comparatively less explored in in vivo models.

Evidence from previous studies demonstrates that both *Trigonella foenum-graecum* (fenugreek) and *Trigonella corniculata* exert significant antihyperlipidemic effects through distinct molecular and metabolic targets. *T. foenum-graecum* seed supplementation has been shown to lower serum total cholesterol, LDL (low-density lipoprotein), and triglycerides while increasing HDL, effects attributed to enhanced fiber and saponin content, upregulation of hepatic LDL receptor (LDLR) gene expression, and improved liver antioxidant status [9,10,11]. Several experimental models confirm these benefits; fenugreek administration markedly increases LDLR expression, facilitating greater hepatic uptake and clearance of circulating LDL cholesterol and overall lipid reduction [12,13]. Additional mechanisms reported include modulation of bile acid metabolism, inhibition of cholesterol absorption, and protection against diet-induced hepatic tissue damage. Synergistic responses have also been observed in polyherbal formulations containing *T. corniculata*, which further improve lipid profiles by targeting oxidative pathways, preserving endothelial function, and reducing lipid peroxidation [12,14]. Collectively, these findings establish *T. foenum-graecum* and *T. corniculata* as promising bioactive agents for hyperlipidemia management, with targets spanning cholesterol biosynthesis, receptor-mediated clearance, antioxidant defense, and histopathological liver protection [15,16].

In this study, we aimed to assess the comparative antioxidant potential and investigate the hypolipidemic potential of *T. foenum-graecum* and *T. corniculata* for managing dyslipidemia through natural, plant-based bioactive components. *T. foenum-graecum*, commonly known as “methi” in Pakistan, is primarily valued for its seeds and leaves. The seeds are used as condiments and supplements, while the leaves serve as vegetables for cooking [17]. This plant exhibits lipid-lowering therapeutic properties, reducing serum triglyceride, total cholesterol, and low- and very-low-density cholesterol levels, while enhancing LDLR gene expression and HDL-C levels [14,18]. The bioactive components found in various parts of this plant contribute to cholesterol metabolism and synthesis, indicating potential therapeutic effects in dyslipidemia [19]. These components include polyphenols, flavonoids, lysine, L-tryptophan, alkaloids, amino acids, proteins, saponins, vitamin C, niacin, and potassium [20].

*T. corniculata*, commonly referred to as “Kasuri methi” in Pakistan, is primarily esteemed for its unique aroma and nutrient-dense leaves. Originating from the Kasur district of Pakistan, Kasuri methi, or cultivated fenugreek, is well regarded in traditional medicine [20,21]. This plant is rich in nutrients such as proteins, fibers, calcium, potassium, phosphorus, magnesium, and iron, all of which contribute to its health benefits. Additionally, *T. corniculata* is abundant in powerful antioxidants and bioactive compounds, which may aid in the treatment and prevention of various diseases [22]. As suggested by previous study findings, both species are rich in essential bioactive compounds, including saponins, flavonoids, alkaloids, tannins, and polyphenols, which contribute to a wide array of pharmacological effects such as antioxidant, anti-inflammatory, antihyperlipidemic, and hepatoprotective activities. Previous studies have highlighted their lipid-lowering and glycemic-regulating potentials, indicating that these plants can effectively modulate lipid metabolism, enhance antioxidant defenses, and prevent oxidative damage associated with metabolic disorders [13,14,15,16,17,18]. Given the increasing global prevalence of hyperlipidemia and related cardiovascular diseases, exploring *T. foenum-graecum* and *T. corniculata* offers a promising, natural, and cost-effective therapeutic alternative. Therefore, the current study aims to evaluate the antioxidant and hypolipidemic potential of *Trigonella foenum-graecum* and *Trigonella corniculata*. Comparison of hypolipidemic, antioxidant, and histopathological effects of both of the above plants under high-fat-diet-induced hyperlipidemia strengthens their potential role as functional foods or nutraceuticals for preventing metabolic and cardiovascular complications.

## 2. Materials and Methods

### 2.1. Raw Material Procurement

The *T. foenum-graecum* and *T. corniculata* seeds were procured from a reputable local market in the Layyah district. After collection, the seeds were thoroughly cleaned to remove contaminants, including dirt and debris. They were then dried in the shade at room temperature to ensure moisture-free seeds. Once dried, the seeds were finely ground using a laboratory grinding machine and stored in labeled zip-lock bags at room temperature. This preparation was maintained to ensure quality and prevent degradation of bioactive compounds in the seeds. All laboratory procedures were conducted in the Nutrition Lab, Department of Human Nutrition, BZU, Multan.

- Inclusion Criteria:

Uncontaminated *T. foenum-graecum* and *T. corniculata* seeds of superior quality were selected to ensure reliable and consistent results.

- Exclusion Criteria:

Seeds that showed signs of contamination were excluded to obtain meaningful and trustworthy findings.

### 2.2. Phytochemical Analysis

The phytochemical analysis of *T. foenum-graecum* and *T. corniculata* was performed according to Asad et al., 2021 with slight modifications [15]. First, aqueous, ethanol, ethyl acetate, methanol, and acetone extracts of both varieties of ground fenugreek seeds were prepared. A quantity of 10 g of powder from each sample in 100 mL (1:10) *w*/*v* of solvent in a conical flask was used to prepare extracts, which were kept in an orbital shaker (Model No: SK-O330-Pro, BIOBASE, Jinan, China) for 36 h. Whatman No. 1 filter paper was used to filter the extract solutions. Solvent evaporation was carried out using a rotary evaporator (Model No: BK-RE-1A, BIOBASE, Jinan, China). The extracts obtained were stored in plastic bottles at a temperature of 4 °C. Subsequently, antioxidant analyses were conducted, including by using DPPH and FRAP methods, and quantitative phytochemical analysis was performed by TPC assessments.

#### 2.2.1. Chemical and Reagents

DPPH was procured from Sigma-Aldrich (Merk, Darmstadt, Germany). Other chemicals used were sodium carbonate (Thermo Fisher Scientific, Waltham, MA, USA), gallic acid (Thermo Fisher Scientific, USA), acetone (Thermo Fisher Scientific, Mumbai, India), ethanol (Thermo Fisher Scientific, India), methanol (Thermo Fisher Scientific, India), ethyl acetate (VWR International, Bangalore, India), and Hematoxylin and Eosin (Abcam, Cambridge, UK). All chemical and reagents used in this study were analytical grade.

#### 2.2.2. TPC Analysis

TPC was assessed using the Folin–Ciocalteu method as outlined by Akbari et al. (2020), employing the Folin–Ciocalteu reagent and analytical grade standard gallic acid [23]. Initially, 0.2 mL of the extract was combined with 0.2 mL of the Folin–Ciocalteu reagent and left at room temperature in the dark for a few minutes. Subsequently, 0.6 mL of sodium carbonate (20% Na2CO3) was added, and the reaction mixture was kept in the dark for two hours. The absorbance of the sample was measured at 765 nm using a spectrophotometer (model number: BK-UV1600G, BIOBASE, Jinan, China). The findings were expressed as milligrams of gallic acid equivalent (GAE) per gram of the extract (mg GAE/g).

#### 2.2.3. DPPH Radical Scavenging Activity Analysis

For the DPPH analysis, a DPPH solution in methanol was prepared following the procedure outlined by Chaubey et al. (2018), which involves dissolving 24 mg of DPPH in 100 mL of methanol and storing it at −20 °C until needed [24]. Then, 2 mL of this solution was added to 0.5 mL of seed extract, mixed, and placed in the dark until further use. The absorbance at 517 nm was measured using a UV Visible Spectrophotometer (Infitek, SP-MUV6000, Jinan, China). The same procedure was performed for all extracts from both samples.

#### 2.2.4. FRAP Analysis

FRAP activity was estimated according to the capacity of the antioxidant compounds to reduce the Fe^3+^-TPTZ complex (colorless) to the Fe^2+^-tripyridyltriazine complex (blue) at low pH. For this assay, 3.995 µL of the sample extract was mixed with 5 mL of the FRAP reagent, including 300 mmol/L sodium acetate buffer, 10 mL of TPTZ in 40 mmol/L HCl, and 20 mmol/L FeCl_3_.6H_2_O. The following reaction was measured at an absorbance of 593 nm. The above procedure was performed on all samples, and the readings obtained were noted [24].

### 2.3. In Vivo Assay

This in vivo study was designed to investigate the hypolipidemic potential of two varieties of fenugreek seeds, *T. foenum-graecum* and *T. corniculata*.

#### 2.3.1. Animal Procurement

Thirty mature (8–10 weeks old; 25–30 g weight) male white albino mice were obtained from UVAS, Lahore.

#### 2.3.2. Research Design

This study was designed as a randomized control trial. Mice were randomly divided into six groups, each with five mice with dietary interventions as mentioned in Table 1. The study design included six experimental groups to assess the effects of *T. corniculata* and *T. foenum-graecum* on hyperlipidemia under a standard basal diet (SBD). Group G0 served as the positive control receiving only the standard basal diet, while G1 was the negative control also receiving SBD and hyperlipidemia. Groups G2 and G3 received SBD supplemented with 400 mg/kg body weight of TC and TFG, respectively, to evaluate the effects of moderate dosing. Groups G4 and G5 were administered higher doses of 800 mg/kg body weight of TC and TFG, respectively, to assess dose-dependent responses.

#### 2.3.3. Efficacy Studies and Housing of Rats

The environmental conditions included a room temperature of 25 ± 2 °C and a light and dark period of 12/12 h. Before the different experimental procedures were initiated, all animals were acclimatized for at least one week. During this time, they had ad libitum access to both water and standard laboratory diet food.

#### 2.3.4. Standard Basal Diet Preparation

The mice were fed a standard basal diet during the study. This diet included a defined number of fenugreek varieties. The percentage composition of the diet is mentioned in Table 2.

#### 2.3.5. Treatment Diet Preparation (Pellet Formation)

Dyslipidemia was induced in the mice by feeding them a 40% high-fat diet (HFD). After inducing dyslipidemia, the treatment diet was prepared by admixing sample seed powders of *T. foenum-graecum* and *T. corniculata* at doses of 400 and 800 mg/kg body weight/day, respectively, with the standard basal diet. The mixture was then formed into ball pellets and administered to the respective groups orally for approximately 4 weeks.

#### 2.3.6. Animal Slaughtering Protocols

Ketamine hydrochloride at a dose of 70 mg/kg B.W was used intraperitoneally to anesthetize the mice. The mice were then sacrificed through dissection. Blood Serum Tubes and EDTA tubes were used to collect blood samples. For serum isolation, the collected samples were then centrifuged (at 3000 rpm) for 5 min [25] and stored at −20 °C in Eppendorf tubes. The serum was used for the examination of biochemical parameters, that is, liver function and lipid profile tests, and blood samples were evaluated for hematological analysis. The liver and heart tissues were histopathologically examined.

#### 2.3.7. Ethical Considerations

This study was conducted in strict adherence to the ethical guidelines set forth by the Bioethical Committee of Bahauddin Zakariya University, Multan, under committee number 89/04-2024. Ethical approval was obtained before the commencement of the study, and all experiments followed the Declaration of Helsinki’s principles. The welfare of the animals was paramount throughout the study, with necessary precautions taken to minimize discomfort and ensure humane treatment.

#### 2.3.8. Weight Estimation

The food and water consumption of each mouse was determined daily, while the body weights of the mice were measured weekly during the experiments. Similarly, organ weights were measured using a digital weight balance after dissection. Organs examined were the liver, right and left kidneys, heart, and spleen. The mentioned organs were procured, washed, and preserved in formalin solution, followed by histopathological examination [14].

#### 2.3.9. Liver Function Test

The liver function test (LFT) was performed as a global check-up on the status of the liver, using the assessed automated serum analyzer. LFT can determine significant parameters, ascertain correct hepatic function, and increase liver enzymes. These serum enzymes include ALT, AST, ALP, and bilirubin [16].

#### 2.3.10. Blood Lipid Profiling

Blood lipid profiling was performed to investigate the hypolipidemic effects of *T. foenum graecum and T. corniculata.* The centrifuged blood serum was analyzed using a biochemical analyzer (BC400, CONTEC Medical Systems Co., Ltd., Qinhuangdao, China) to determine lipid levels [13].

#### 2.3.11. Hematological Examination

Hematological parameters were investigated to determine hypolipidemic effects. Blood samples were collected into EDTA tubes and the blood samples analyzed for hematology parameters using a YSTE120C, Guangdong China hematology analyzer [25,26].

#### 2.3.12. Histopathological Examination

To evaluate the hypolipidemic effects of T. *foenum graecum* and T. *corniculata* on dyslipidemia, histopathological studies of liver and heart sections were performed. Following anesthesia administration to mice, the liver and heart were dissected, and the obtained organ tissues were placed in 10% buffered formalin at room temperature overnight [21]. The tissues were trimmed to very thin sections and placed on glass slides, and specific stains were applied to the tissue to enhance tissue architecture. These sections were then observed under a light microscope, and pictures were taken for further scrutiny, which provided histopathological analysis with special reference to inflammation and necrosis.

### 2.4. Statistical Analysis

The obtained results were analyzed using Statistix 8.1 computer software for statistical analysis to ensure the accuracy of these obtained results. In the current experimental design, the independent variables were analyzed using analysis of variance (ANOVA) to identify sources of variance between groups followed by Tukey’s post hoc HSD test.

## 3. Results

Antioxidant potential analysis revealed significant differences in the antioxidant capacity between the two fenugreek seed varieties (*T. foenum-graecum* (TFG) and *T. corniculata* (TC)) using different solvents, as mentioned in Table 3. The analysis of variance (ANOVA) results showed highly significant effects of the solvent, plant variety, and their interaction on the DPPH, TPC, and FRAP values (all with *p*-values less than 0.01), as indicated by the sum of squares and degrees of freedom (DF). The LSD all-pairwise comparisons test revealed detailed comparisons, indicating that TFG generally exhibited stronger antioxidant activity than TC. Specifically, TFG showed higher DPPH values with ethanol (88.48 ± 1.44) and acetone (86.37 ± 0.17), suggesting superior radical scavenging activity.

**Figure 1:**
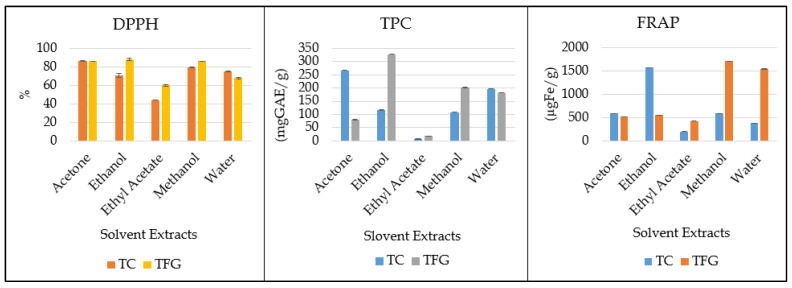
Graphical representation of antioxidant capacities of different TC and TFG solvent extracts. (In DPPH figure, % mean percentage of inhibition of DPPH free radical).

The antioxidant potential of the seeds, as measured by DPPH radical scavenging activity, was ranked in the following order: ethanol (TFG) > acetone (TC) > acetone (TFG) > methanol (TC) > methanol (TFG) > water (TC) > water (TFG) > ethanol (TC) > ethyl acetate (TFG) > ethyl acetate (TC), as mentioned in Figure 1. In terms of total phenolic content (TPC), TFG also outperformed TC, with ethanol (328.28 ± 0.10) and methanol (201.87 ± 0.37) extractions indicating a higher phenolic content. The TPC analysis shows the solvents’ effectiveness in descending order as follows: ethanol (TFG) > acetone (TC) > methanol (TFG) > water (TC) > water (TFG) > acetone (TFG) > ethanol (TC) > methanol (TC) > ethyl acetate (TFG) > ethyl acetate (TC). The FRAP assay showed that the methanolic extract of TFG had higher antioxidant potential than the other extracts. FRAP values showed that methanol had higher free radical scavenging activity. The FRAP results ranked the solvents from most to least effective as follows: methanol (TFG) > ethanol (TFG) > ethanol (TC) > acetone (TC) > methanol (TC) > acetone (TFG) > water (TFG). In conclusion, this study confirms that *T. foenum-graecum* imparts higher antioxidant activity in most of the solvents used than *T. corniculata*. Among all the solvents used for the extraction of the antioxidants from TFG, ethanol and methanol revealed higher DPPH, TPC, and FRAP values.

**Figure 2:**
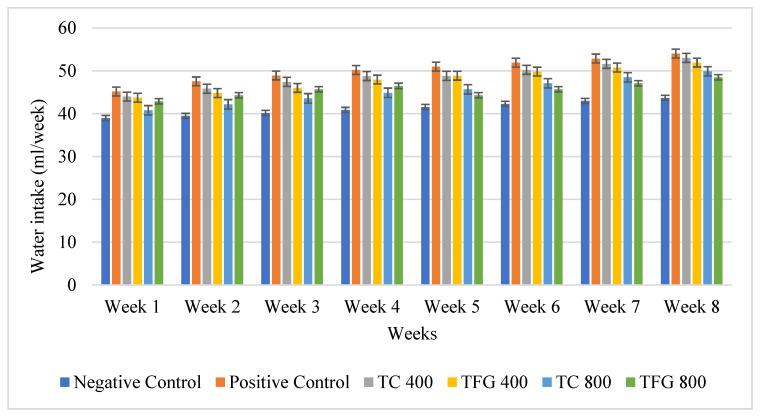
Graphical representation of the water intake of mice per week.

Water intake of the mice over an 8-week study duration is depicted in the bar graph (Figure 2). Water intake across all groups showed a pattern of fluctuations, with some weeks showing slight decreases followed by increases. Overall, water intake showed a slight increase across all groups during the dyslipidemia induction phase, while the negative control group showed a comparatively lower intake. After dyslipidemia induction, the treatment period started, and the treatment groups showed comparatively less water intake than the positive group, but water intake gradually increased with time in all groups. The highest water intake was generally observed in the later weeks (Week 7 and Week 8), suggesting that the demand for water might increase as age progresses, which could be indicative of a higher metabolic rate or different physiological needs. In conclusion, water intake varied slightly from week to week but generally stayed within a consistent range for each group.

**Figure 3:**
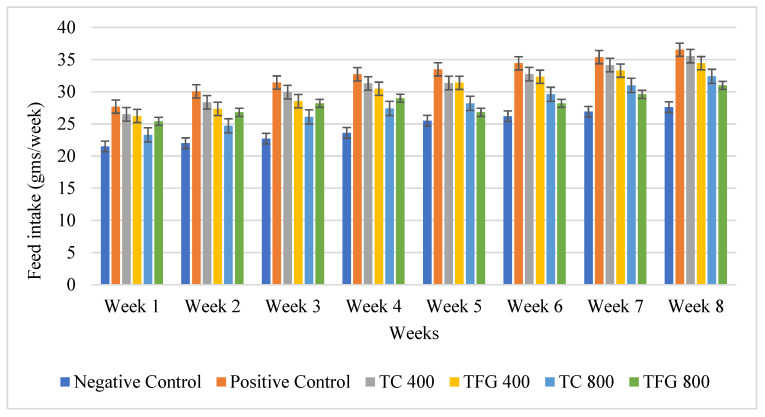
Graphical representation of the feed intake of mice per week.

The feed intake of the mice in all groups was noted daily and the data are depicted in the bar graph (Figure 3) as average intake of feed per mouse of all the groups. Among all the groups, the positive control group consistently showed the highest feed intake across all weeks, while the negative control group showed the lowest feed intake. For the treatment groups, both plant seeds showed similar trends with minor variations. The feed intake in groups 4 and 5 (*T. foenum-graecum* and *T. corniculata* at 800 mg/kg BW/day) was comparatively the lowest among the treated groups and was close to that of the negative control. However, there was an overall increase in all groups over time. The error bars indicate the standard deviation, reflecting the variability within each group.

This study revealed a significant reduction in body weight across groups treated with both TFG and TC compared to the positive control group (dyslipidemic mice without treatment). The highest reduction was observed at a higher dose (800 mg/kg BW/day), indicating a dose-dependent response. Plant type also played a significant role, with TFG showing a slightly more pronounced effect than TC. From this, it can be deduced that bioactive constituents of TFG have a better impact on metabolic activities that control body weight, as mentioned in Table 4. The LSD test results show that the body weight of the positive control group G_0_ (37.10 ± 0.53 g) was significantly higher than that of all other groups, with the negative control G1 having the lowest weight (30.30 ± 0.36 g). Moderate doses of TC (G_2_; 33.67 ± 0.87 g) and TFG (G_3_; 32.20 ± 0.2 g) resulted in intermediate body weights, while higher doses (G_4_ and G_5_) showed slight weight reductions compared to moderate doses. Similarly, organ weights (liver, heart, kidneys, spleen, lungs) were highest in G0 and decreased progressively with treatment doses, with G5 generally showing the lowest values (e.g., liver weight 1.31 ± 0.01 g, heart weight 0.16 ± 0.02 g). Shared superscript letters indicate groups that do not differ significantly at *p* > 0.05.

The effect of dose on triglyceride (TG) levels was highly significant (*p* < 0.001), indicating that different doses had a considerable impact on TG levels. The effect of plant type was not significant (*p* > 0.05), suggesting that TFG and TC had similar effects on TG levels. However, the interaction between dose and plant type was significant (*p* < 0.05), implying that the combined effects of dose and plant type influenced TG levels. The positive control group (G0) had the highest TG level (85.67 ± 3.51 mg/dL), while the negative control group (G_1_) had the lowest (46.00 ± 1.53 mg/dL). TG levels significantly decreased with both doses of TC and TFG, with the lowest levels observed in G1, followed by G5 (62.00 ± 1.0 mg/dL), as displayed in Table 5.

Total cholesterol (TC-c) also showed a highly significant dose effect (*p* < 0.001), but the plant effect was not significant (*p* > 0.05). The interaction between dose and plant type was not significant (*p* > 0.05). Both doses of TC and TFG reduced TC levels significantly, with G_4_ and G_5_ showing similar reductions (122.00 ± 0.56 mg/dL and 122.00 ± 1.92 mg/dL, respectively). Low-density lipoprotein (LDL) levels were significantly affected by dose (*p* < 0.001) and plant type (*p* < 0.05). The interaction between dose and plant type was also significant (*p* < 0.01), indicating that both factors significantly influenced LDL-c levels. LDL levels decreased significantly with treatment, with G_5_ showing a notable reduction (83.86 ± 0.80 mg/dL). For high-density lipoprotein (HDL), the dose had a highly significant effect (*p* < 0.001), and plant type also showed a highly significant effect (*p* < 0.01). However, the interaction between dose and plant type was not significant (*p* > 0.05). Both TC and TFG increased HDL levels, with G_5_ showing a significant improvement (22.69 ± 0.58 mg/dL). Very-low-density lipoprotein (VLDL) levels were significantly affected by the dose (*p* < 0.001). The plant effect was not significant (*p* > 0.05); however, the interaction between the dose and plant type was significant (*p* < 0.05). G_0_ had the highest VLDL-c level (17.13 ± 0.702 mg/dL) and G_1_ had the lowest (9.20 ± 0.31 mg/dL). Both doses of TC and TFG reduced VLDL-c levels, with G_5_ again showing a significant decrease (12.40 ± 0.2 mg/dL).

The liver function of white albino mice treated with different doses of *T. foenum-graecum* and *T. corniculata* seeds was evaluated through various biochemical parameters including bilirubin, alanine transaminase (ALT), aspartate transaminase (AST), and alkaline phosphatase (ALP). The results were analyzed using ANOVA and LSD all-pairwise comparisons test, as mentioned in Table 6. For bilirubin, the dose effect was highly significant (*p* < 0.001), indicating that different doses had a significant impact on bilirubin levels. The results also revealed that the type of plant had a significant impact, suggesting that TFG and TC have an impact on bilirubin levels. The main effects of dose and plant type were highly significant (*p* < 0. 01), depicting that the dose and plant type greatly affected bilirubin levels. In TFG and TC groups, treatment groups recorded low bilirubin levels, the lowest being in G5 (0.23 ± 0.01 mg/dL) followed by G_4_ (0.27 ± 0.010 mg/dL).

Regarding the dose effect, statistical analysis showed a significant effect for ALT at *p* < 0. 001, whereas the plant effect was not significant. The results of this study also indicated that the effect of the interaction between dose and plant type was not significant (*p* > 0. 05). ALT levels were also lower with treatment, especially in G_5_: 176.33 ± 2.08 IU/L. For AST, the dose effect was highly significant (*p* < 0.001), whereas the plant effect was significant (*p* < 0.05). The interaction between dose and plant type was not significant (*p* > 0.05). AST levels decreased significantly with treatment, with G_5_ showing a large reduction (188.0 ± 4.0 IU/L). For ALP, the dose effect was highly significant (*p* < 0.001). The plant effect was significant (*p* < 0.05); however, the interaction between the dose and plant type was not significant (*p* > 0.05). ALP levels decreased significantly with treatment, with G_5_ showing a notable reduction (148.6 ± 2.52 IU/L) followed by G_4_ (157.33 ± 2.52 IU/L).

The hematological profile includes red blood cell count, hemoglobin, hematocrit, mean corpuscular volume, mean corpuscular hemoglobin, mean corpuscular hemoglobin concentration, WBCs, lymphocytes, neutrophils, comprising monocytes, eosinophils, and basophils from differential WBC counts, and platelets. Blood samples were also tested using a fully automated hematology analyzer, as analyzed in Table 7. Different doses of *T. foenum-graecum* and *T. corniculata* seeds had a significant effect on the hematological parameters of white albino mice, such as white blood cells (WBCs), lymphocytes, neutrophils, mixed cells, and platelets. The collected data were statistically analyzed using analysis of variance and the least significant difference pairwise comparison method.

Similarly, we found the dose effect to be highly significant for all parameters: WBCs, lymphocytes, neutrophils, mixed cell count, and platelets; therefore, different doses had an impact on their concentration (*p* < 0.001). The plant effect was also highly significant (*p* < 0.001) for WBCs, lymphocytes, mixed cell count, and platelets, but not for neutrophils (*p* > 0.05), suggesting that TFG and TC had different effects on WBCs, lymphocytes, mixed cell count, and platelets levels. The interaction between dose and plant type was highly significant for platelets (*p* < 0.001) and significant (*p* <0.05) for lymphocytes. However, it was not significant (*p* > 0.05) for WBC, neutrophils, or mixed cell count. The positive control group (G_0_) had the highest WBC count (11.65 ± 0.19 × 10^3^/µL), while the negative control group (G_1_) had the lowest (7.13 ± 0.12 × 10^3^/µL). WBC levels were significantly lower in groups treated with TFG and TC, with G_5_ showing a significant reduction (8.51 ± 0.31 × 10^3^/µL). Lymphocytes, neutrophils, and mixed cells levels were all significantly lowered in treated groups, with G_5_ showing a maximum reduction of 6.10 ± 0.10 × 10^3^/µL, 1.48 ± 0.17 × 10^3^/µL, and 0.92 ± 0.05 × 10^3^/µL, respectively. Platelet count was significantly reduced in the positive group and increased varyingly in the treatment groups, with G_5_ showing the maximum increase over time, i.e., 960.7 ± 4.16 × 10^3^/µL.

One-way ANOVA of the hematological profile of RBCs in white albino mice was used to evaluate the effect of treatments on various blood parameters. Thus, the type of plant and the dose of treatment have significant effects on all the observed parameters, as displayed in Table 8. Significant differences in red blood cell count, hemoglobin, hematocrit, mean corpuscular volume, mean corpuscular hemoglobin, and mean corpuscular hemoglobin concentration were observed. The findings of this study showed that the types of plants and their concentrations affected these factors. The variance analysis demonstrated that the interaction between dose and plant type significantly influenced hematocrit (*p* < 0.001), whereas other parameters remained unaffected. This indicates that the dose–plant interaction is not broadly applicable, but specifically affects the proportion of blood incorporated by RBCs. The pairwise comparison test highlights the differences in hematological profiles among various groups (G_0_ to G_5_). Among the treatment groups, *T. foenum-graecum* and *T. corniculata*, both administered at higher doses of 800 mg/kg, BW/day, exhibited a more effective response, with TFG showing the most pronounced effect. All parameters, including RBC count, hemoglobin, hematocrit, mean corpuscular volume, mean corpuscular hemoglobin, and mean corpuscular hemoglobin concentration varied significantly across the groups, with the highest values observed in G_5_, followed by G4. This indicates that both the treatment dose and plant type significantly influence the hematological parameters in white albino mice, with the interaction between these factors particularly affecting hematocrit levels. These findings underscore the importance of treatment variables in influencing blood health.

**Figure 4:**
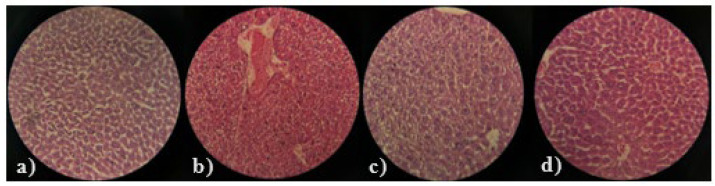
Histopathological image of liver tissue; (**a**) TC 400 mg/kg B.W (**b**) TC 800 mg/kg B.W (**c**) TFG 400 mg/kg B.W (**d**) TFG 800 mg/kg B.W.

**Figure 5:**
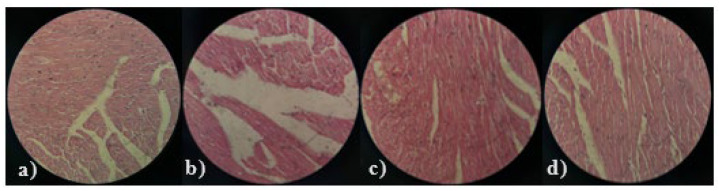
Histopathological image of the heart tissue; (**a**) TC 400 mg/kg B.W (**b**) TC 800 mg/kg B.W (**c**) TFG 400 mg/kg B.W (**d**) TFG 800 mg/kg B.W.

Histopathological examination of liver and heart tissues was performed to further analyze the effects of the seeds of both varieties of fenugreek, that is, *T. foenum-graecum* and *T. corniculata*, both at different doses of 400 and 800 mg/kg B.W. The histopathological interpretation of the liver and heart tissues of all treated groups are mentioned in Figure 4 and Figure 5, respectively. In the hepatic parenchyma of TC (400 mg/kg B.W), the hepatocytes were arranged in cords in most areas. Sinusoidal spaces were prominent, with prominent round, centrally placed nuclei. At some places, the blood vessels were congested, and prominent perivascular cuffing was observed. In the parenchyma, scattered inflammatory cells were observed in a few areas, along with the presence of round vacuoles. Overall, the severity of degenerative and necrotic changes was minimal. In the hepatic parenchyma of TC (800 mg/kg B.W), prominent cytoplasmic vacuolation with small round vacuoles was observed. In some areas, the hepatocytes showed coagulative necrosis, along with the absence of sinusoidal spaces. The blood vessels were congested. However, the severity of the inflammatory response was minimal. Pyknotic nuclei of hepatocytes were prominent in certain areas. Perivascular cuffing was absent in most areas compared to the group TC 400.

The hepatocytes of TFG (400 mg/kg B.W) showed coagulated necrosis along with clear round cytoplasmic vacuolation. The inflammatory response was minimal. However, slight congestion was observed in the inner parenchyma. At some places, sinusoidal spaces were absent, indicating cellular swelling of hepatocytes. No prominent perivascular cuffing or inflammatory response was observed. In the TFG (800 mg/kg B.W) group, the hepatocytes were arranged in clear cords with well-defined sinusoidal spaces. No prominent degenerative or inflammatory responses were observed in the parenchyma. The hepatocytes exhibited a more or less normal architectural structure. However, slight congestion was observed in a few blood vessels.

In the myocardium of the TC 400 mg/kg B.W group, no prominent degenerative or necrotic changes were observed. The muscle bundle fibers were intact. Slight detachment of the muscle fiber was present in a few places. The blood vessels were congested, and areas of hemorrhagic response were observed. However, the degenerative and inflammatory responses were minimal. In the myocardium of the TC 800 mg/kg B.W group, the muscle bundle fibers were intact. No prominent necrotic changes were observed. Congestive blood vessels and a few hemorrhagic areas were also present. No prominent inflammatory response was observed in the myocardium.

In the myocardium of the TFG 400 mg/kg B. W group, there were congested blood vessels and areas of hemorrhagic response. In a few places, there was detachment of muscle fibers, along with the presence of inflammatory cells. In certain areas, pink pigmentation was observed in individual muscle fibers. In the myocardium of the TFG 800 mg/kg B. W group, the muscle bundle fibers were intact. No inflammatory response was observed. Congestion was minimal. No prominent hemorrhagic response was noted, but slight vacuolation between the individual muscle fibers was observed in a few places.

## 4. Discussion

Dyslipidemia, a major cardiovascular risk factor, is characterized by increased total cholesterol (TC), low-density lipoprotein cholesterol (LDL-C), VLDL-C, and triglycerides (TG) with decreased HDL-C levels [27]. These changes can lead to atherosclerosis, arrhythmias, and stroke. While *T. foenum-graecum* and *T. corniculata* are common in Pakistan, research on *T. corniculata’s* therapeutic potential remains limited. This study investigated the antioxidant potential of *T. foenum-graecum* (TFG) and *T. corniculata* (TC) seeds at 400 and 800 mg/kg B.W daily doses.

Among the antioxidant properties, TFG generally exhibits stronger antioxidant activity than TC. Specifically, TFG showed higher DPPH values with ethanol (88.48 ± 1.44) and acetone (86.37 ± 0.17), suggesting superior radical scavenging activity. During the in vivo study to assess the hypolipidemic effects of the two varieties of fenugreek seeds, different parameters were assessed. Overall, water and feed intake showed a slight increase across all groups during the dyslipidemia induction phase, whereas the negative control group showed a comparatively lower intake. After dyslipidemia induction, the treatment period started, and the treatment groups showed comparatively lesser intakes than the positive group, but gradually increased over time in all groups. The highest intakes were generally observed in the later weeks (Week 7 and Week 8), suggesting that the demand for water and feed might increase as the age progresses, which could be indicative of a higher metabolic rate or different physiological needs [14,15].

The results revealed the hypolipidemic effect of TFG and TC at different doses on lipid profile in the dyslipidemia mice model. Administration of 800 mg/kg TFG caused the greatest reduction in TC, LDL-C, VLDL-C, and TG levels as well as an increase in HDL-C. Following this, TC at a dose of 800 mg/kg demonstrated notable hypolipidemic effects, albeit to a lesser extent than TFG. At a dose of 400 mg/kg, both TFG and TC exhibited reduced effects; however, 400 mg/kg TFG yielded better outcomes than 400 mg/kg TC. This aligns with earlier research indicating that fenugreek may be more effective in managing lipid levels [28,29]. The study observed that 800 mg/kg TFG elevated HDL-C levels while reducing TC, LDL-C, VLDL-C, and TG levels. These effects are likely due to the high levels of saponins, flavonoids, and fibers, which are known to inhibit cholesterol absorption and facilitate cholesterol absorption [17,30]. Administering *Trigonella foenum-graecum* and *Trigonella corniculata* seeds led to significant enhancements in liver function parameters in dyslipidemic mice, with higher doses generally producing favorable results.

The study by Abd El-Megeid et al. (2025) demonstrated that fenugreek seed powder and its oil significantly improved lipid profiles and reduced hyperlipidemia-related body and organ weight changes in rats, supporting fenugreek’s potential as a dietary intervention for dyslipidemia [13]. Jahan et al. (2024) found that supplementation of high-fat-diet-fed rats with fenugreek powder ameliorated metabolic syndrome by reducing oxidative stress and inflammation, highlighting fenugreek’s multifaceted protective mechanisms against diet-induced metabolic disorders [14]. Asad et al. (2021) reported fenugreek’s ameliorative effects against copper oxide nanoparticle-induced toxicity in fish (*Oreochromis mossambicus*), showing that its antioxidant and protective properties extend across species and toxic insults [15]. Collectively, these studies corroborate the role of fenugreek in improving lipid metabolism, reducing oxidative damage, and supporting organ health in hyperlipidemia and related metabolic conditions. Notable interactions between dose and plant type were observed for bilirubin, underscoring the potential of these plants to manage liver function when used at optimal doses. These findings suggest that both TFG and TC possess hepatoprotective properties, making them beneficial for treating liver-related disorders. One study reported increased ALP and AST activity in the treated group, whereas plant supplementation significantly reduced ALP and AST levels. The study indicated that the plants protect the liver tissue from alcohol-induced toxicity in rats, which is attributed to the pharmacological and antioxidant activities of the plant [31,32,33].

The seeds of *T. foenum-graecum* and *Trigonella corniculata* caused notable changes in the blood profile of white albino mice. Different concentrations led to significant differences in WBCs, lymphoid cells, neutrophilic cells, mixed cells, and platelets. Nagamma et al. (2023) demonstrated that TFG and TC influence hematological parameters and could potentially be beneficial in treating certain conditions [34]. All parameters, including RBC count, Hb level, HCT, MCV, MCH, and MCHC, varied significantly among the groups, with the highest values observed in G5, followed by G4. This indicates that both the dosage and the type of plant used significantly affect the hematological parameters of white albino mice, with a particular impact on hematocrit levels.

Both TC and TFG seeds caused dose-dependent histopathological changes in the liver and heart tissues. Higher doses of TFG appeared to maintain normal tissue architecture and intact heart muscle fibers. However, the recovery of the inflammatory changes was not as significant in *T*. *corniculata* as in *T*. *foenum-graecum*. Both *Trigonella foenum-graecum* and *Trigonella corniculata* demonstrated good antioxidant potential. Both have shown potential in the management of dyslipidemia by improving various parameters, but TFG 800 mg/kg has a better overall histopathological profile than that reported in previous studies [33,34]. The current study’s findings align well with the existing literature on *Trigonella* species by demonstrating significant reductions in body and organ weights associated with hyperlipidemia following supplementation with *T. foenum-graecum* (TFG) and *T. corniculata* (TC). Consistent with Bakhtiar et al. (2024), who highlighted the lipid-regulating and antioxidant capacities of *Trigonella* seeds due to bioactive compounds such as diosgenin and trigonelline, our study observed that TFG and TC effectively decreased LDL cholesterol and liver weight, suggesting reduced lipid accumulation and liver stress [35,36]. These effects further corroborate those of Fatima et al. (2022), who emphasized the antioxidant and anti-inflammatory potential of *T. foenum-graecum* extracts, which are crucial in mitigating oxidative damage and inflammation in metabolic diseases [37]. The observed improvements in heart and kidney weights in the current study echo the cardioprotective and nephroprotective roles discussed by various previous study reinforcing the therapeutic relevance of fenugreek in metabolic syndrome [9,36,38]. Additionally, the dose-dependent effects noted in parallel by Lohvina et al. (2021) indicate that solvent extraction and phytochemical concentration critically influence Trigonella’s efficacy [39]. Overall, the current outcomes not only validate fenugreek’s traditional and pharmaceutical applications but also provide robust biochemical and histopathological evidence to support its use as a natural treatment for hyperlipidemia and liver health preservation.

Limitations of this study include the use of animal models, which may limit direct translation of results to humans, and the evaluation of only two dose levels, restricting the full understanding of dose–response relationships without in vivo exact molecular confirmation. Prospects involve conducting clinical trials to validate efficacy and safety in humans, exploring longer-term administration effects, standardizing fenugreek extract formulations for consistent bioactive content, and elucidating molecular pathways underlying the lipid-lowering and hepatoprotective effects of *T. foenum-graecum* and *T. corniculata*. Advances in biotechnology, such as mutant line development, may also enhance yield and phytochemical profiles and optimize therapeutic potential.

## 5. Conclusions

A comparative study of *Trigonella foenum-graecum* (TFG) and *Trigonella corniculata* (TC) showed free radical scavenging activity and increased ferric-reducing antioxidant power in various solvents. Both TFG and TC improved lipid profiles in dyslipidemic mice by lowering total cholesterol, LDL-c, VLDL-c, and triglycerides, while raising HDL-c. Histopathological analysis showed TFG offered superior protection, with minimal degeneration in liver and heart tissues at 400 and 800 mg/kg doses, and nearly normal liver structure and intact myocardial fibers at the higher dose.

In conclusion, both *T. foenum-graecum* and *T. corniculata* have demonstrated significant antioxidant and hypolipidemic properties, with *T. foenum-graecum* showing superior effects on lipid profiles, liver function, and histopathological outcomes. These findings suggest that both plant varieties could serve as functional foods or nutraceuticals for managing dyslipidemia, with potential clinical applications in the prevention and treatment of cardiovascular diseases. Future clinical trials are necessary to confirm their safety and efficacy in human populations.

## Figures and Tables

**Table 1:** Experimental design for in vivo study.

Groups	Treatments	Dosing Regimen
G_0_	Positive control	Disease + standard basal diet
G_1_	Negative control	Standard basal diet
G_2_	TC (400 mg/kg B.W)	SBD supplemented with TC 400 mg/kg/day
G_3_	TFG (400 mg/kg B.W)	SBD supplemented with TFG 400 mg/kg/day
G_4_	TC (800 mg/kg B.W)	SBD supplemented with TC 800 mg/kg/day
G_5_	TFG (800 mg/kg B.W)	SBD supplemented with TFG 800 mg/kg/day

SBD: standard basal diet. TFG: Trigonella foenum-graecum. TC: Trigonella corniculate.

**Table 2:** Ingredients of standard diet.

Ingredients	Percentage
Corn starch	68%
Casein	11%
Wheat Bran	7%
Cellulose	5%
Soybean oil	4%
Mineral Mix	2%
Vitamin Mix	1%

**Table 3:** Phytochemical analysis of *T. foenum-graecum* and *T. corniculata* in different solvents.

Plant	Solvent	DPPH (%)	TPC (mgGAE/g)	FRAP (µgFe/g)
TC	Acetone	86.77 ± 0.42 ^a^	266.52 ± 0.08 ^b^	584.8 ± 0.30 ^c^
	Ethanol	70.96 ± 2.27 ^cd^	116.73 ± 0.71 ^f^	1576.5 ± 0.40 ^b^
	Ethyl Acetate	44.13 ± 0.04 ^f^	6.44 ± 0.19 ^j^	199.6 ± 1.69 ^g^
	Methanol	79.87 ± 0.53 ^b^	108.02 ± 0.02 ^g^	584.7 ± 1.14 ^c^
	Water	75.37 ± 0.55 ^bc^	196.60 ± 0.12 ^d^	375.4 ± 1.99 ^f^
TFG	Acetone	86.37 ± 0.17 ^a^	80.07 ± 0.82 ^h^	522.5 ± 0.25 ^d^
	Ethanol	88.48 ± 1.44 ^a^	328.28 ± 0.10 ^a^	550.0 ± 2.89 ^d^
	Ethyl Acetate	60.16 ± 0.71 ^e^	17.23 ± 0.48 ^i^	425.1 ± 0.25 ^e^
	Methanol	86.34 ± 0.05 ^a^	201.87 ± 0.37 ^c^	1712.9 ± 1.11 ^a^
	Water	68.04 ± 0.97 ^d^	181.78 ± 0.08 ^e^	1549.0 ± 1.58 ^b^

Means having same letters do not differ significantly (*p* values >0.05). TFG: *Trigonella foenum-graecum*. TC: *Trigonella corniculate*.

**Table 4:** All pairwise comparison test of body and organ weight for plant extract versus dose.

Parameters	G_0_	G_1_	G_2_	G_3_	G_4_	G_5_
Body Weight (g)	37.10 ± 0.53 ^a^	30.30 ± 0.36 ^d^	33.67 ± 0.87 ^b^	32.20 ± 0.2 ^c^	32.20 ± 0.36 ^c^	31.07 ± 0.60 ^d^
Liver (g)	1.475 ± 0.03 ^a^	1.39 ± 0.02 ^b^	1.38 ± 0.04 ^bc^	1.36 ± 0.06 ^c^	1.33 ± 0.05 ^d^	1.31 ± 0.01 ^d^
Heart (g)	0.19 ± 0.01 ^a^	0.17 ± 0.03 ^b^	0.17 ± 0.01 ^bc^	0.17 ± 0.01 ^c^	0.17 ± 0.01 ^d^	0.16 ± 0.02 ^d^
Right Kidney (g)	0.14 ± 0.04 ^a^	0.12 ± 0.02 ^c^	0.12 ± 0.04 ^cd^	0.11 ± 0.05 ^de^	0.11 ± 0.03 ^ef^	0.11 ± 0.01 ^f^
Left Kidney (g)	0.15 ± 0.01 ^a^	0.13 ± 0.02 ^b^	0.13 ± 0.02 ^bc^	0.13 ± 0.01 ^cd^	0.13 ± 0.01 ^de^	0.13 ± 0.01 ^e^
Spleen (g)	0.11 ± 0.00 ^a^	0.11 ± 0.02 ^b^	0.10 ± 0.02 ^bc^	0.10 ± 0.02 ^cd^	0.10 ± 0.03 ^de^	0.09 ± 0.06 ^e^
Lungs (g)	0.41 ± 0.04 ^a^	0.38 ± 0.07 ^b^	0.37 ± 0.01 ^bc^	0.37 ± 0.30 ^cd^	0.36 ± 0.03 ^de^	0.36 ± 0.02 ^e^

Means having same letters do not differ significantly (*p* values > 0.05), ANOVA test followed by Tukey’s post hoc HSD test. G_0_; Disease + SBD. G_1_; SBD. G_2_; Disease + SBD + TC 400 mg/kg/day. G_3_; Disease + SBD + TFG 400 mg/kg/day. G_4_; Disease + SBD + TC 800 mg/kg/day. G_5_; Disease + SBD + TFG 800 mg/kg/day.

**Table 5:** All pairwise comparisons test of lipid profile for plant extract versus dose.

Lipid Test Parameters	G_0_	G_1_	G_2_	G_3_	G_4_	G_5_
TG (mg/dL)	85.67 ± 3.51 ^a^	46.00 ± 1.53 ^e^	71.0 ± 1.0 ^b^	68.00 ± 1.0 ^c^	64.67 ± 0.58 ^d^	62.00 ± 1.0 ^d^
TC-c (mg/dL)	143.0 ± 2.42 ^a^	112.00 ± 0.75 ^d^	129.00 ± 0.71 ^b^	126.67 ± 0.68 ^b^	122.00 ± 0.56 ^c^	122.00 ± 1.92 ^c^
HDL-c (mg/dL)	16.23 ± 0.57 ^e^	27.51 ± 0.9 ^a^	18.24 ± 0.91 ^d^	19.87 ± 0.79 ^c^	21.59 ± 0.41 ^b^	22.69 ± 0.58 ^b^
LDL-c (mg/dL)	105.96 ± 0.32 ^a^	72.66 ± 0.64 ^e^	93.46 ± 0.54 ^b^	89.98 ± 0.98 ^c^	85.44 ± 1.08 ^d^	83.86 ± 0.80 ^d^
VLDL-c (mg/dL)	17.13 ± 0.702 ^a^	9.20 ± 0.31 ^e^	14.20 ± 0.2 ^b^	13.60 ± 0.2 ^c^	12.93 ± 0.12 ^d^	12.40 ± 0.2 ^d^

Means having same letter do not differ significantly (*p* values > 0.05), ANOVA test followed by Tukey’s post hoc HSD test, TG: Triglyceride, TC-c: Total Cholesterol. G_0_; Disease + SBD. G_1_; SBD. G_2_; Disease + SBD + TC 400 mg/kg/day. G_3_; Disease + SBD + TFG 400 mg/kg/day. G_4_; Disease + SBD + TC 800 mg/kg/day. G_5_; Disease + SBD + TFG 800 mg/kg/day.

**Table 6:** All pairwise comparisons test of liver profile for plant extract versus dose.

Liver Blood Test Parameters	G_0_	G_1_	G_2_	G_3_	G_4_	G_5_
Bilirubin (mg/dL)	0.42 ± 0.01 ^a^	0.22 ± 0.01 ^f^	0.35 ± 0.010 ^c^	0.31 ± 0.01 ^d^	0.27 ± 0.010 ^e^	0.23 ± 0.010 ^f^
ALT (IU/L)	194.0 ± 2.0 ^a^	167.0 ± 3.79 ^e^	189.0 ± 1.0 ^b^	183.67 ± 1.53 ^c^	178.33 ± 3.06 ^d^	176.33 ± 2.08 ^d^
AST (IU/L)	279.3 ± 5.03 ^a^	184.0 ± 5.3 ^e^	229.67 ± 4.7 ^b^	216.0 ± 4.58 ^c^	195.33 ± 5.03 ^d^	188.0 ± 4.0 ^de^
ALP (IU/L)	191.0 ± 4.58 ^a^	139.0 ± 2.0 ^e^	178.33 ± 2.1 ^b^	173.0 ± 2.0 ^b^	157.33 ± 2.52 ^c^	148.6 ± 2.52 ^d^

Means having same letters do not differ significantly (*p* values > 0.05), Alanine transaminase (ALT), Aspartate transaminase (AST), Alkaline phosphatase (ALP), ANOVA test followed by Tukey’s post hoc HSD test. G_0_; Disease + SBD. G_1_; SBD. G_2_; Disease + SBD + TC 400 mg/kg/day. G_3_; Disease + SBD + TFG 400 mg/kg/day. G_4_; Disease + SBD + TC 800 mg/kg/day. G_5_; Disease + SBD + TFG 800 mg/kg/day.

**Table 7:** All pairwise comparisons test of hematological profile (WBCs and platelets count) of white albino mice.

Hematological Parameters	G_0_	G_1_	G_2_	G_3_	G_4_	G_5_
WBC (10^3^/μL)	11.65 ± 0.19 ^a^	7.13 ± 0.12 ^g^	10.68 ± 0.36 ^c^	9.910 ± 0.21 ^d^	9.35 ± 0.2 ^e^	8.51 ± 0.31 ^f^
Lymphocytes (10^3^/μL)	7.40 ± 0.1 ^a^	5.06 ± 0.15 ^f^	7.13 ± 0.12 ^b^	6.83 ± 0.06 ^c^	6.43 ± 0.08 ^d^	6.10 ± 0.10 ^e^
Neutrophils (10^3^/μL)	2.34 ± 0.2 ^a^	1.34 ± 0.08 ^d^	2.08 ± 0.10 ^b^	1.88 ± 0.1 ^bc^	1.71 ± 0.04 ^c^	1.48 ± 0.17 ^d^
MXD (10^3^/μL)	1.91 ± 0.15 ^a^	0.92 ± 0.06 ^d^	1.46 ± 0.16 ^b^	1.19 ± 0.07 ^c^	1.23 ± 0.12 ^c^	0.92 ± 0.05 ^de^
Platelets (10^3^/μL)	698.3 ± 5.86 ^g^	1062.0 ± 6.08 ^a^	782.3 ± 12.7 ^e^	809.0 ± 10.39 ^d^	878.0 ± 2.0 ^c^	960.7 ± 4.16 ^b^

Means having same alphabets do not differ significantly (*p* values > 0.05), WBC: White blood cell, MXD: Mixed cell count, ANOVA test followed by Tukey’s post hoc HSD test. G_0_; Disease + SBD. G_1_; SBD. G_2_; Disease + SBD + TC 400 mg/kg/day. G_3_; Disease + SBD + TFG 400 mg/kg/day. G_4_; Disease + SBD + TC 800 mg/kg/day. G_5_; Disease + SBD + TFG 800 mg/kg/day.

**Table 8:** LSD all-pairwise comparisons test of hematological profile (RBCs) of white albino mice.

Parameters	G_0_	G_1_	G_2_	G_3_	G_4_	G_5_
RBC (10^6^/µL)	7.103 ± 0.1 ^g^	10.89 ± 0.21 ^a^	8.55 ± 0.21 ^f^	8.98 ± 0.11 ^e^	9.45 ± 0.21 ^d^	10.08 ± 0.23 ^c^
Hb (g/dL)	12.97 ± 0.12 ^g^	14.96 ± 0.12 ^a^	13.53 ± 0.15 ^e^	13.86 ± 0.16 ^d^	14.27 ± 0.15 ^c^	14.63 ± 0.15 ^b^
HCT (%)	33.93 ± 0.69 ^f^	46.73 ± 0.26 ^a^	41.63 ± 0.06 ^d^	41.83 ± 0.12 ^d^	43.33 ± 0.55 ^c^	44.62 ± 0.56 ^b^
MCV (fl)	49.38 ± 0.08 ^e^	50.92 ± 0.53 ^ab^	49.76 ± 0.09 ^de^	49.89 ± 0.04 ^cde^	49.99 ± 0.06 ^cd^	50.34 ± 0.29 ^bc^
MCH (pg)	12.64 ± 0.1 ^e^	17.13 ± 0.74 ^a^	13.44 ± 0.08 ^d^	14.20 ± 0.01 ^c^	14.81 ± 0.04 ^c^	15.58 ± 0.52 ^b^
MCHC (g/dL)	31.70 ± 0.53 ^f^	35.01 ± 0.25 ^a^	32.86 ± 0.32 ^d^	33.36 ± 0.20 ^cd^	33.80 ± 0.17 ^bc^	34.18 ± 0.33 ^b^

Means having same alphabets do not differ significantly (*p* values > 0.05), ANOVA test followed by Tukey’s post hoc HSD test, RBC: Red blood cell, Hb: Hemoglobin, HCT: Hematocrit, MCV: Mean corpuscular volume, MCH: Mean corpuscular hemoglobin, MCHC: Mean corpuscular hemoglobin concentration. G_0_; Disease + SBD. G_1_; SBD. G_2_; Disease + SBD + TC 400 mg/kg/day. G_3_; Disease + SBD + TFG 400 mg/kg/day. G_4_; Disease + SBD + TC 800 mg/kg/day. G_5_; Disease + SBD + TFG 800 mg/kg/day.

## Data Availability

The original contributions presented in the study are included in the article, further inquiries can be directed to the corresponding authors.
